# Supplementary material for: Chaenomeles sinensis Extract Ameliorates Ovalbumin-Induced Allergic Rhinitis by Inhibiting the IL-33/ST2 Axis and Regulating Epithelial Cell Dysfunction
Source: Foods. 2024 Feb 18;13(4):611. doi: 10.3390/foods13040611 (PMC10888344; doi:10.3390/foods13040611)
Supplement: Supplementary file 1 [file foods-13-00611-s001.zip › foods-2852936-supplementary.pdf]

Supplementary material for

Chaenomeles sinensis extract ameliorates ovalbumin-induced allergic rhinitis by inhibiting the IL-33/ST2 axis and regulating epithelial cell dysfunction

Juan Jin[1*], Yan Jing Fan[1,2*] Thi Van Nguyen[1], Zhen Nan Yu[1], Chang Ho Song[1,3], So-Young Lee[4,5], Hee Soon Shin[4,5], Ok Hee Chai[1,3]

[1] Department of Anatomy, Jeonbuk National University Medical School, Jeonju, 54896, Jeonbuk, Korea

[2] School of Medicine, Liaocheng University, Liaocheng, Shandong, People's Republic of China.

[3] Institute for Medical Sciences, Jeonbuk National University Medical School, Jeonju, 54896, Jeonbuk, Korea

[4] Department of Food Biotechnology, University of Science and Technology (UST), Daejeon 34113, Korea

[5] Division of Food Functionality Research, Korea Food Research Institute (KFRI), Wanju 55365, Korea

Correspondence: Ok Hee Chai, Department of Anatomy and Institute for Medical Sciences, Jeonbuk National University Medical School, Jeonju, 54896, Jeonbuk, Korea. Tel: +82-63-2703109, Fax: +82-63-2749880. Email: okchai1004@jbnu.ac.kr.

* These authors contributed equally to this work.

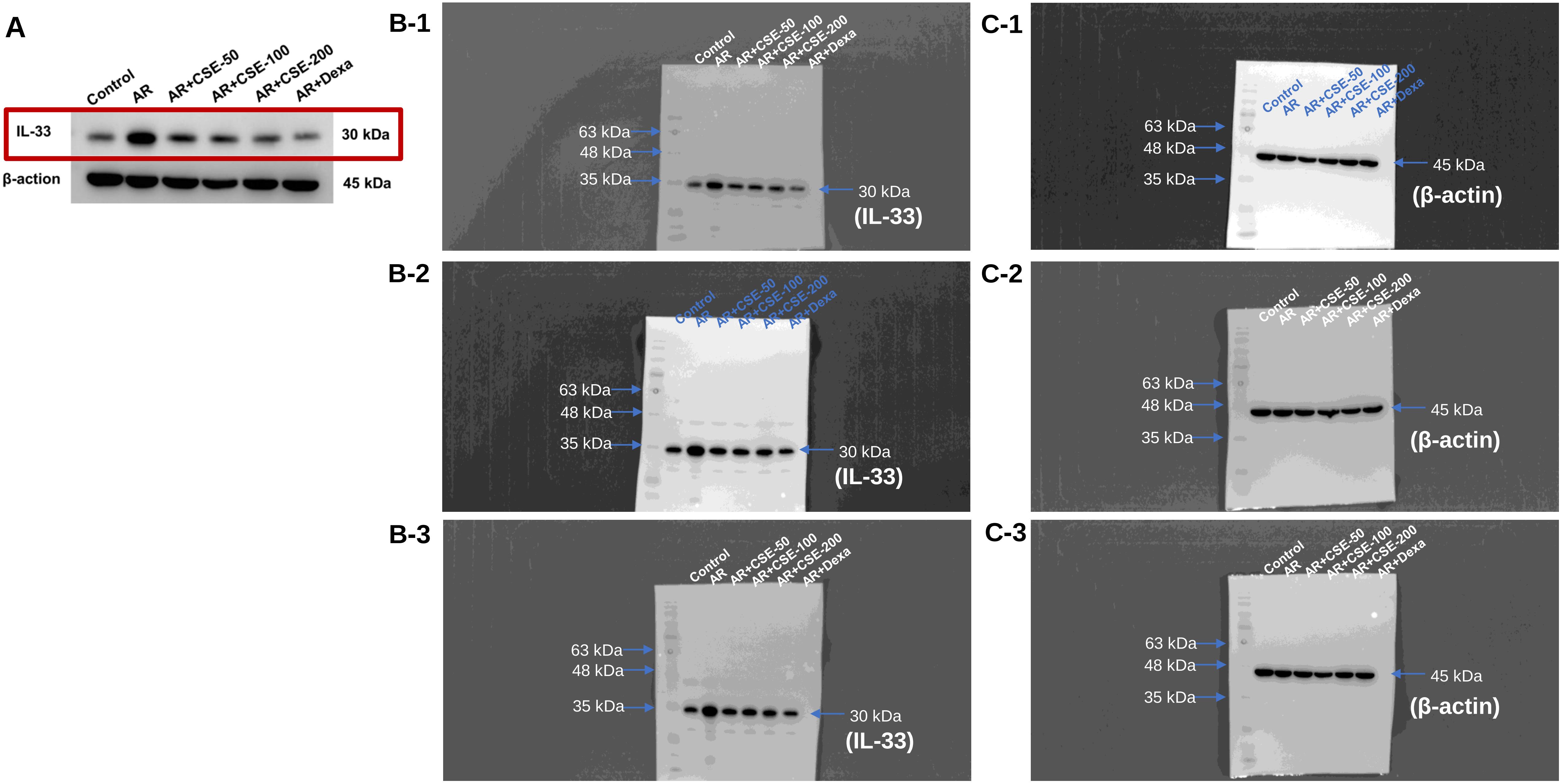

Figure S1. Full-length gels and blots of IL-33 signaling-related proteins in lung tissues (A) Western blot data. Original Western blot picture of (B) IL-33 and (C) β-actin. Membrane C was stripped from membrane B.

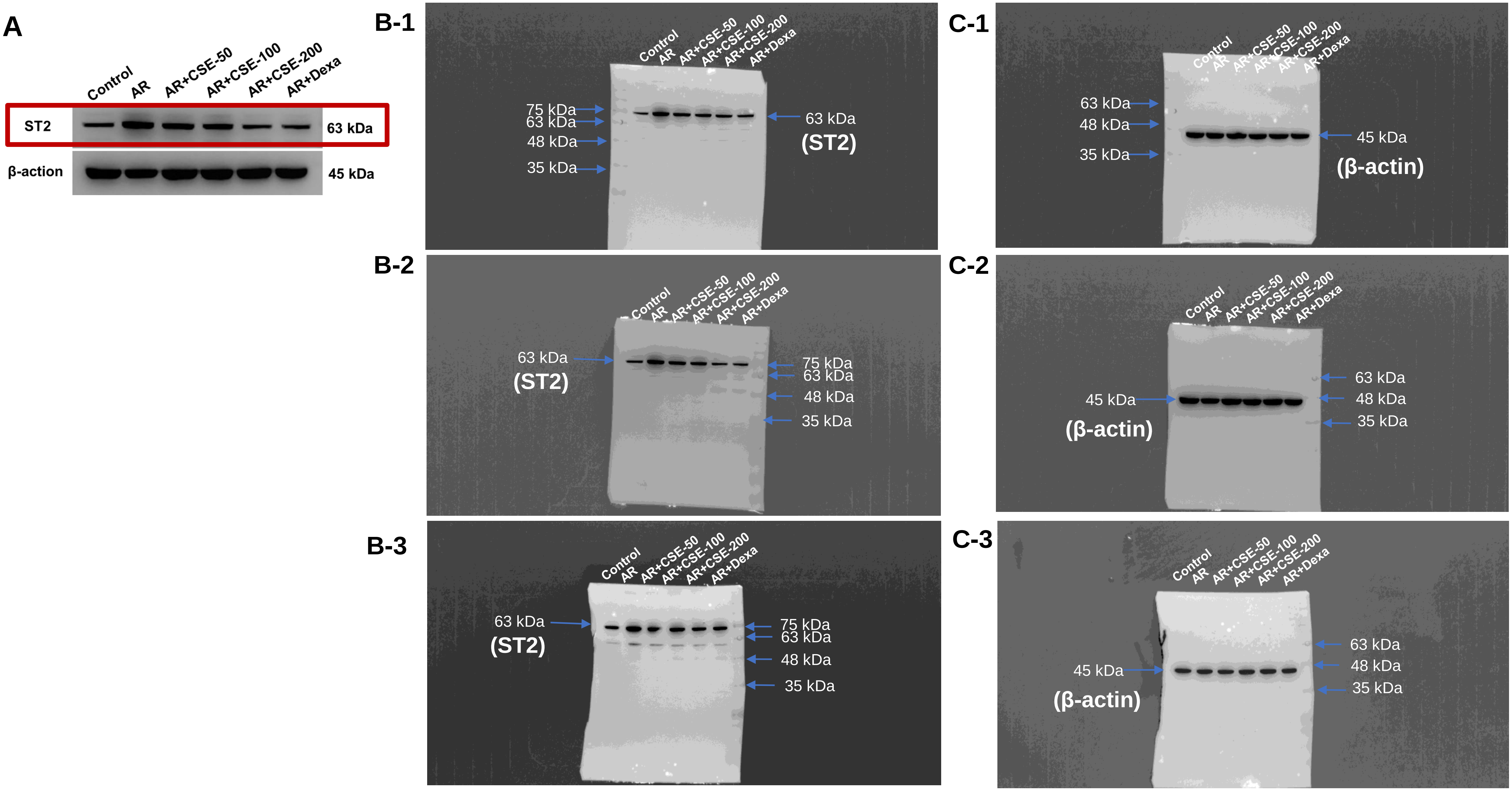

Figure S2. Full-length gels and blots of ST2 signaling-related proteins in lung tissues (A) Western blot data. Original Western blot picture of (B) IL-33 and (C) β-actin. Membrane C was stripped from membrane B.
